# Low Prostaglandin E_2_ but High Prostaglandin D_2_, a Paradoxical Dissociation in Arachidonic Acid Metabolism in Aspirin-Exacerbated Airway Disease: Role of Airway Epithelium

**DOI:** 10.3390/jcm13237416

**Published:** 2024-12-05

**Authors:** César Picado, Liliana Machado-Carvalho, Jordi Roca-Ferrer

**Affiliations:** 1 Institut d’Investigacions Biomèdiques August Pi I Sunyer (IDIBAPS), University of Barcelona, 08907 Barcelona, Spain; jrocaf@recerca.clinic.cat; 2 Centro de Investigaciones en Red de Enfermedades Respiratorias (CIBERES), 28029 Madrid, Spain; 3 Department of Biology and Environment, School of Life Sciences and Environment, University of Trás-os-Montes and Alto Douro (UTAD), 5000-801 Vila Real, Portugal; lilianac@utad.pt; 4 Center for Research and Technology of Agro-Environmental and Biological Sciences (CITAB-UTAD), University of Trás-os-Montes and Alto Douro (UTAD), 5000-801 Vila Real, Portugal

**Keywords:** alarmins, airway epithelium, aspirin-exacerbated respiratory disease, asthma, innate immunity, mast cells, prostaglandin E2, prostaglandin D2

## Abstract

In patients with aspirin-exacerbated respiratory disease (AERD), there is disparate regulation of prostaglandin E2 (PGE_2_) and prostaglandin D_2_ (PGD_2_). Both prostanoids are synthesised by cyclooxygenase 1 (COX-1) and cyclooxygenase 2 (COX-2). However, while the basal synthesis of PGE_2_ tends to decrease, that of PGD_2_ increases in patients with AERD. Furthermore, both behave differently in response to the inhibitory action of NSAIDs on COX-1: PGE_2_ levels decrease while PGD_2_ increases. Increased PGD_2_ release correlates with nasal, bronchial, and extra-pulmonary symptoms caused by aspirin in AERD. The proposed hypothesis establishes that the answer to this paradoxical dissociation can be found in the airway epithelium. This is based on the observation that reduced COX-2 mRNA and/or protein expression is associated with reduced PGE_2_ synthesis in cultured fibroblast and epithelial cells from AERD compared to patients with asthma who are aspirin-tolerant and healthy subjects. The low production of PGE_2_ by the airway epithelium in AERD results in an excessive release of alarmins (TSLP, IL-33), which in turn contributes to activating group 2 innate lymphoid cells (ILC2s) and PGD_2_ synthesis by mast cells and eosinophils. Aspirin, by further increasing the diminished PGE_2_ regulation capacity in AERD, leads to respiratory reactions associated with the surge in PGD_2_ from mast cells and eosinophils. In summary, the downregulation of COX-2 and the subsequent low production of PGE_2_ by airway cells account for the apparently paradoxical increased production of PGD_2_ by mast cells and eosinophils at the baseline and after aspirin provocation in patients with AERD. A better understanding of the role of the airway epithelium would contribute to elucidating the mechanism of AERD.

## 1. Introduction

Aspirin-exacerbated respiratory disease (AERD), known in Europe as nonsteroidal anti-inflammatory drug (NSAID)-exacerbated respiratory disease (N-ERD), is a clinical syndrome involving the upper and lower respiratory tracts. It is characterised by the association of chronic rhinosinusitis with nasal polyps (CRSwNPs), asthma, and hypersensitivity to NSAIDs [1].

Our understanding of the pathophysiology of AERD has increased over the past four decades; however, there is still no definitive explanation of the mechanism leading to this distinctive clinical syndrome. The evidence so far suggests that altered regulation of the cyclooxygenase (COX) and 5-lipoxygenase (5-LO) pathways of arachidonic acid (AA) is involved in AERD [1]. Nevertheless, our present understanding of the disturbed AA metabolism in AERD is incomplete, and numerous unknowns remain to be elucidated [1].

One of the intriguing observations in AERD is the disparate regulation of prostaglandin E_2_ (PGE_2_) and prostaglandin D_2_ (PGD_2_). Both prostanoids are initially synthesised from the action of the cyclooxygenase 1 (COX-1) and cyclooxygenase 2 (COX-2) enzymes, subsequently following specific enzymatic pathways. However, while basal synthesis of PGE_2_ tends to decrease, that of PGD_2_ is increased in patients with AERD. Furthermore, both behave differently in response to the inhibitory action of NSAIDs on COX-1: PGE_2_ levels decrease while PGD_2_ increases. Increased PGD_2_ release temporally occurs with the surge in cysteinyl leukotriene (Cys-LT) release and correlates with the onset and course of nasal, bronchial, and extra-pulmonary symptoms caused by NSAIDs in AERD [1].

To the best of our knowledge, no attempt has been made to find a potential explanation for this apparently paradoxical dissociated release of PGE_2_ and PGD_2_. We hypothesise that understanding the mechanism underlying this dissociation will advance knowledge of AERD pathophysiology.

A literature search of PubMed was conducted for papers published on the topic of this manuscript. The search words of AERD, airway epithelium, arachidonic acid, asthma, aspirin, chronic rhinosinusitis, and cyclooxygenase were used.

## 2. Arachidonic Acid Metabolism

**Figure 1:**
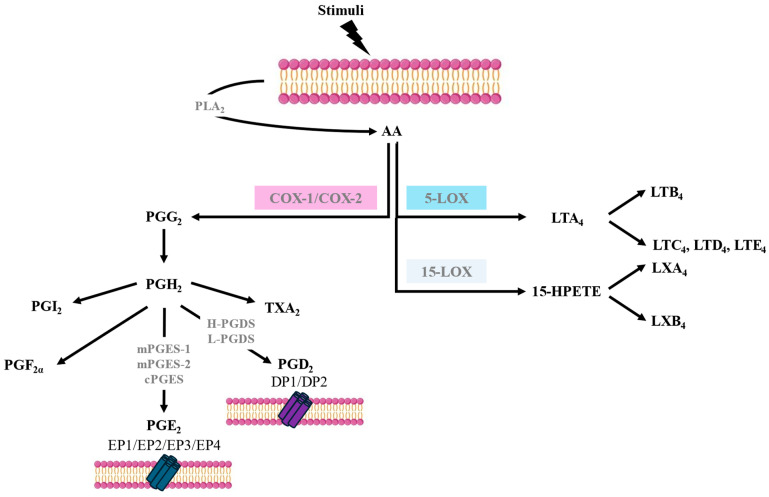
Arachidonic acid metabolism pathways.

AA is a polyunsaturated fatty acid that is released from cell membrane phospholipids, primarily through the action of phospholipase A2 (PLA2). Various stimuli, such as immune signals or cell injury, can trigger the activation of PLA2. AA metabolism involves several pathways such as COX, also known as prostaglandin-endoperoxide synthase (PTGS), 5-lipoxygenase (5-LOX), and 15-lipoxygenase (15-LOX), leading to the synthesis of different bioactive lipid mediators. The biosynthesis of eicosanoids occurs in a stepwise fashion and involves several enzymes, not just a single enzyme [2] (Figure 1). AA metabolism through the COX pathway is a crucial biochemical process that plays a central role in various physiological functions and the regulation of inflammation. The COX pathway is responsible for the synthesis of prostanoids. There are two main isoforms of COX—COX-1 and COX-2—each encoded by a different gene but having 60% amino acid identity. COX-1 is constitutively expressed in many tissues, and it is involved in maintaining normal physiological functions. The enzyme largely remains stable; however, small increases in its expression can occur in response to stimulation with some cytokines, hormones, and growth factors. COX-2, on the other hand, is inducible, and its expression can be increased dramatically after the exposure of cells to cytokines, bacterial lipopolysaccharides, or growth factors; therefore, it is often upregulated during inflammation. However, low constitutive levels of COX-2 can also be found in some tissues, such as the vascular endothelium, pulmonary epithelial cells, and some kidney cells [2].

The *COX-1* gene, once expressed, is sustained, whereas *COX-2* is usually transiently expressed after induction. The action of both COXs on AA leads to the formation of prostaglandin H2 (PGH_2_), which is subsequently converted into prostaglandins (PGs), thromboxanes, and prostacyclin, with the contribution of different specific terminal synthases [2] (Figure 1).

PGE_2_ synthesis via the COX pathways involves three PGE_2_ synthases: microsomal PGE_2_ synthase-1 (mPGES-1), microsomal PGE_2_ synthase-2 (mPGES-2), and cytosolic PGE_2_ synthase (cPGES). Like COX-2, mPGES-1 expression is induced by pro-inflammatory cytokines and growth factors and contributes to the regulation of pain, fever, and inflammatory responses. mPGES-1 preferentially couples with COX-2 to synthesise PGE_2_. In contrast, the expression and activity of mPGES-2 are constitutive and, therefore, not significantly upregulated during inflammation. cPGES is mainly constitutive and not induced by inflammatory stimuli and couples more efficiently with COX-1 than with COX-2 in generating PGE_2_. There are cell types that are major contributors to PGE_2_ synthesis, such as epithelial cells, fibroblasts, vascular endothelial cells, smooth muscular cells, and macrophages [2,3] (Figure 1).

There are four PGE_2_ receptors (EP1-EP4) that possess varied and opposing actions due to coupling via G-proteins to several signal transduction pathways that effect Ca^2+^ mobilisation and stimulation or the inhibition of adenylate cyclase, which regulates cyclic adenosine monophosphate (cAMP) synthesis. EP2 and EP4 receptor activation increases cAMP levels, resulting in anti-inflammatory and smooth muscle relaxant effects, while EP1 receptor activation mediates Ca^2+^ mobilisation and induces smooth muscle contraction. Finally, the EP3 receptor inhibits adenylate cyclase, resulting in lowering cAMP levels and thereby inhibiting smooth muscle relaxation [4,5] (Figure 1).

PGD_2_ is released mainly by mast cells and eosinophils in response to IgE-mediated and non-IgE-mediated activation and is synthesised through the enzymatic activity of prostaglandin D synthases (PGDSs). There are two main PGDSs: hematopoietic prostaglandin D synthase (H-PGDS) and lipocalin-type prostaglandin D synthase (L-PGDS). H-PGDS is primarily expressed in mast cells, Th2 lymphocytes, eosinophils, and macrophages. H-PGDS plays a crucial role in the synthesis of PGD_2_ during allergic and non-allergic reactions and inflammation. L-PGDS is predominantly expressed in the central nervous system, where it is involved in various neurological functions, including sleep regulation and pain perception [2,5].

PGD_2_ signals via two receptors termed DP1 and DP2. DP1 is expressed in goblet cells in the nasal and colonic mucosa, nasal serous glands, vascular endothelium, basophils, eosinophils, Th2 cells, and dendritic cells (DCs). DP1 stimulation activates adenylate cyclase, resulting in increased intracellular cAMP. DP2, also known as a chemoattractant receptor-like molecule expressed on Th2 cells (CRTH2), is expressed in eosinophils, basophils, CD4 Th2, and CD8 Tc2 lymphocytes. DP2 signalling in eosinophils augments their release from bone marrow and increases their degranulation and respiratory burst. In contrast to DP1 signalling, the activation of DP2 reduces intracellular cAMP and, therefore, would facilitate mediator release by mast cells [5,6] (Figure 1).

## 3. Dissociated PGE_2_ and PGD_2_ Release in AERD

NSAIDs work by inhibiting the activity of COX enzymes, thus blocking the synthesis of prostanoids and exerting analgesic effects. However, the use of COX inhibitors can have side effects as the balance of prostanoids is critical for normal physiological functions [7].

The COX pathway is common for both PGE_2_ and PGD_2_ synthesis [2]. When COX-1 is inhibited by aspirin, PGH_2_ synthesis is affected. Since both PGE and PGD synthases utilise PGH_2_ as a substrate, the reduction in PGH_2_ levels due to COX-1 inhibition by aspirin leads to a decrease in both PGE_2_ and PGD_2_ synthesis in healthy subjects [2]. However, as mentioned above, in patients with AERD, the production of both prostanoids appears to be dissociated at the baseline and after aspirin exposure [1]. These differences suggest that the synthesis of these prostanoids diverges somewhere along the AA metabolism pathways in patients with AERD (Figure 1).

Next, we will analyse what is known about COX pathway regulation in AERD to find an explanation for this PGE_2_/PGD_2_ production dissociation.

### 3.1. Cyclooxygenase 1 in AERD

Aspirin-related inhibition of COX-1 plays a central role in AERD; however, very little research has focused on the mechanisms underlying the enzyme’s exquisitely heightened sensitivity to blockage caused by low-dose aspirin in patients with AERD, compared to aspirin-tolerant subjects. It is generally assumed that the concentration of COX-1 remains stable under normal conditions; however, two to fourfold increases in expression can occur in cells exposed to cytokines such as interleukin-1 beta (IL-1β), growth factors, and hormones [8,9]. Several studies have assessed COX-1 regulation using immunohistochemistry methods in nasal polyps and bronchial biopsy samples from subjects with AERD, non-AERD, and controls [10,11,12,13,14]. COX-1 expression was higher in the nasal polyps of patients with AERD and non-AERD, compared to control mucosa, without reaching statistical significance [10,11,12,13,14]. COX-1 upregulation results have also been reported in studies using real-time PCR to measure COX-1 mRNA, with [15] and without [16] statistically significant differences between nasal polyps from patients with AERD and non-AERD compared to nasal mucosa controls.

As COX-1 expression moves within very narrow margins and the number of samples studied was very small, most studies had very low statistical power, leading to a lack of significant differences between inflamed nasal polyps and control tissues [10,11,12,14].

Interestingly, the treatment of nasal polyps with corticosteroids reduces COX-1 expression [17], supporting the notion that inflammation can activate COX-1 expression in the airways.

In contrast to that observed in nasal and bronchial tissue samples, studies carried out in cultured fibroblasts and epithelial cells derived from nasal polyps and bronchial biopsies showed that the lowest amount of COX-1 was found in patients with AERD [18,19]. Moreover, IL-1β-stimulated fibroblasts from patients with AERD did not respond with the expected mild/moderate increase in COX-1 expression, which instead occurred in control nasal mucosa and, to a lesser extent, in individuals with non-AERD [20,21].

As a mild increase in COX-1 expression is the expected response under inflammatory conditions, the lack of response of fibroblasts to an inflammatory stimulus suggests that COX-1 regulation is disturbed in the structural cells of the respiratory mucosa in patients with AERD, which could result in a different response upon NSAID administration in structural and inflammatory cells, as these drugs block PGE_2_ production driven mainly by this enzyme isoform.

### 3.2. Cyclooxygenase 2 in AERD

Under physiological conditions, COX-2 is said to be undetectable or expressed at very low levels in some tissues. COX-2 expression is greatly increased in inflammation and, therefore, according to the dogma, should be over-expressed in the inflamed upper and lower airway tissue of nasal polyps and bronchial asthma [2].

COX-2 expression in patients with AERD has been studied using nasal mucosa, nasal polyp, and bronchial tissue samples. Using immunohistochemical, quantitative PCR, and Western blotting methods, no statistically significant differences in COX-2 expression were found between patients with AERD, patients with non-AERD, and control subjects in some studies [10,11,13,14]. However, COX-2 expression was significantly lower in patients with AERD than in patients with non-AERD in one study [12]; when the analysis was individualised for each specific cell type, the number and percentage of mast cells expressing COX-2 were significantly increased in patients with AERD compared to patients with non-AERD in one study [11], while the number of COX-2-positive epithelial cells was significantly reduced in another [12].

On the other hand, strong expression of COX-2 and H-PGDS mRNA has been observed in nasal polyp mast cells from patients with AERD and non-AERD, but without statistically significant differences [22].

Reduced expression of COX-2 mRNA and/or proteins has been reported in nasal polyps and cultured fibroblasts from patients with AERD and non-AERD with respect to nasal mucosa controls or chronic rhinosinusitis without nasal polyps [15,16,18,19,20,21,23,24,25], with the lowest levels of COX-2 observed in nasal polyp samples and cultured fibroblast and epithelial cells from subjects with AERD [16,21,23,24,25]. mPGES-1, the synthase which preferentially couples with COX-2 to synthesise PGE_2_, was also downregulated in cultured fibroblasts from patients with AERD compared to healthy controls [25].

In contrast to COX-1, treatment with corticosteroids increased COX-2 expression levels in nasal polyps, suggesting that the inflammatory process affecting the airways in patients with nasal polyps and/or asthma may contribute to downregulating COX-2 [26].

### 3.3. Prostaglandin E_2_ and PGE_2_ Receptors in AERD

Numerous in vitro and in vitro studies have assessed PGE_2_ production in patients with AERD, with discrepant results. In vivo studies have reported lower [27] and similar [28,29] PGE_2_ levels in nasal fluid from patients with AERD compared to patients with non-AERD and control individuals. The PGE_2_ levels in bronchoalveolar lavage fluid (BALF) were lower in patients with AERD with respect to subjects with non-AERD [30]. Similar PGE_2_ levels in concentrated exhaled air have been reported in subject with AERD, non- AERD, and controls [31]. Lower urinary PGE_2_ levels [32] and similar urinary levels of the terminal metabolite tetranor PGE-M [33] were reported in patients with AERD with respect to patients with non-AERD in one study [32] and patients with non-AERD and control individuals in another [33].

Numerous ex vivo and in vitro studies have also assessed PGE_2_ generation in patients with AERD. PGE_2_ production by peripheral blood leukocytes was significantly reduced in patients with AERD compared to subjects with non-AERD [34,35] and healthy controls [34]. PGE_2_ concentrations were also lower in nasal polyp tissue [23], cultured epithelial cells [36], and cultured fibroblasts [20,21,22,25] from patients with AERD compared to those from either subjects with non-AERD [20,22,23,25] or control individuals [21,23,26].

The mechanism involved in the downregulation of COX-2 and reduced PGE_2_ production in AERD remains to be fully clarified. Interestingly, some studies have shown that IL-4 and IFNγ could decrease COX-2 expression [37,38]. The combined action of both cytokines can reproduce the COX-2 pathway alterations observed in AERD nasal polyps in healthy nasal mucosa [37]. This suggests that mixed T2 (IL-4) and non-T2 (INF-γ) inflammation may be at the origin of the altered COX-2 regulation and limited PGE_2_ production seen in AERD [37].

As previously mentioned, stimulation of the EP2 receptor by PGE_2_ has anti-inflammatory protective effects. Numerous studies have reported impairment of EP2 mRNA and protein expression in both the upper and lower airways of patients with AERD. In an immunohistochemistry study performed with bronchial biopsies from subjects with AERD, non-AERD, and controls, the authors reported that, compared to individuals with non-AERD, patients with AERD had reduced percentages of T cells, macrophages, mast cells, and neutrophils expressing EP2 [39]. In contrast, a quantitative analysis of EP receptor mRNA expression in peripheral blood mononuclear cells isolated from these patients exhibited no significant differences between the two groups [39]. Downregulated EP2 protein and mRNA expression have also been reported in nasal polyps of patients with AERD compared to individuals with non-AERD [12,40] and control subjects [41]. A similarly altered EP2 regulation has been reported in cultured fibroblasts isolated from AERD nasal polyps compared to those isolated from individuals with non-AERD [21,40] and controls [21,25,41,42]. The altered EP2 expression in AERD results in reduced anti-inflammatory and antiproliferative effects of EP2 signalling in nasal polyp fibroblasts [41,42]. Interestingly, the expression of EP4 has been found augmented in cultured fibroblasts from AERD nasal polyps compared to those isolated from the nasal mucosa of control subjects; however, signalling through EP4 only could partially counteract the deficient EP2-mediated effects [41].

The mechanisms potentially involved in the downregulation of EP2 in AERD include epigenetic factors [42] and *EP2* gene polymorphisms [43].

The reduced production of PGE_2_ associated with deficient EP2 expression could contribute to perpetuating the chronic inflammatory and remodelling processes usually found in the upper and lower airways of patients with AERD [1].

It is worth noting that reduced PGE_2_ production is not exclusive to patients with AERD; it is also present, to a lesser extent, in subjects with non-AERD [22,23,34]. It is unknown whether a threshold exists for the disturbance of COX metabolism and PGE_2_ production beyond which the hypersensitivity reaction to NSAIDs occurs, or if other factors associated with COX pathway dysregulation contribute to NSAID-induced hypersensitive reactions.

Because PGE_2_ production is expected to increase in inflammatory processes, such as nasal polyposis and asthma, the detection of similar PGE_2_ levels in patients with AERD and healthy subjects in nasal fluid, BALF, urine, and concentrated exhaled air samples should be considered an anomaly rather than a normal finding.

In contrast to that observed in AERD, the expression of COX-2 and EP2 in cultured fibroblasts and the PGE_2_ levels in concentrated exhaled air were significantly higher in patients with chronic obstructive pulmonary disease (COPD) than in healthy controls [44,45,46]. Because COPD is also considered an airway inflammatory disease, these observations reinforce the notion that the altered regulation of COX expression and PGE_2_ synthesis in AERD should be considered an aberrant rather than normal response to the expected upregulation of the COX/mPGES-1/PGE_2_ pathway in nasal polyps and bronchial asthma.

Inhibition of COX-1 by aspirin and other NSAIDs results in the blockage of PGE_2_ synthesis, which is commonly considered to be the trigger of asthma exacerbation in patients with AERD [1]. Several studies have assessed the inhibitory effects of aspirin in PGE_2_ production in subjects with AERD, non-AERD, and controls. As expected, aspirin reduced urinary PGE_2_ and PGE-M levels [32,34], as well as PGE_2_ production by peripheral blood leukocytes [35], in individuals without AERD [32,34,35] and healthy controls [35]. Aspirin exerts little inhibitory effects on PGE_2_ production in individuals with AERD [32,34,35]. These observations suggest a reduced sensitivity of COX-1 to the inhibitory effect of aspirin in patients with N-ERD by yet-unknown mechanisms [35].

### 3.4. Prostaglandin D_2_, Mast Cells, and Eosinophils in N-ERD

Mast cells and eosinophils are the major source of PGD_2_, a significant factor in the pathogenesis of allergic diseases, contributing to airway inflammation, mucus production, and bronchoconstriction [1,2]. PGD_2_ is the most prominent COX product released by various in vitro activated human mast cells, with other prostanoids only being synthesised in small amounts [47,48]. PGD_2_ synthesis in mast cells entirely depends on COX-1 activity [47,48]. Human mast cells do not express the COX-2 protein, neither at rest nor after activation [48]. As an expected consequence, in vivo studies in healthy subjects [32] and patients with asthma [32,34] showed that COX-1 inhibition reduced urinary levels of PGD_2_ metabolites; however, selective COX-2 inhibitors had no inhibitory effects in some [48,49] but not all studies [50].

In AERD, there is increased PGD_2_ production, resulting in an elevated urinary level of basal PGD_2_ metabolites (tetranor PGD-M) with respect to subjects without AERD [32,34]. Similarly, baseline concentrations of PGD_2_ in the sputum have been shown to be significantly higher in patients with AERD than in those with non-AERD [51,52]. Furthermore, plasma levels of tetranor PGD-M measured in patients with AERD were also significantly higher than those in patients with non-AERD and healthy control subjects [53]. As other typical markers of mast cell activation, such as tryptase and leukotriene E4, are also elevated in AERD at the baseline, it is generally assumed that most of the PGD_2_ synthesised in these patients should originate from the same cell source [54].

The mechanism responsible for increased PGD_2_ synthesis by mast cells in patients with AERD is currently unclear. COX-1 expression may be moderately upregulated under inflammatory conditions; however, it is unknown whether these minimal changes could contribute to this increase in PGD_2_ synthesis by mast cells in patients with AERD. To the best of our knowledge, there are no studies demonstrating COX-1 upregulation in mast cells from these patients. Other studies reported an increase in the number of mast cells expressing inducible pro-inflammatory COX-2 in patients with AERD [11]. The stimulation of mast cells with IL-33, an alarmin elevated in AERD airways [55], induced a fast and strong increase in COX-2 expression and PGD_2_ production; however, it did not alter COX-1 expression levels [56]. The study also demonstrated that COX-1 is involved in the IL-33-induced activation of PLA2, which is followed by an increased release of AA and the enhanced synthesis of prostanoids such as PGD_2_ [56]. Surprisingly, the inhibition of COX-1 not only decreased the generation of PGD_2_ but also blocked IL-33-induced COX-2 expression [56]. In other studies, COX-2 and H-PGDS mRNA were also upregulated in mast cells from patients with N-ERD and non-NERD, but without significant differences [22,34]. Taken together, these observations suggest that both COX-1 and COX-2 can contribute, through a partially known complex and interdependent mechanism, to the regulation of PGD_2_ synthesis in mast cells under inflammatory conditions in AERD [56].

The COX-1/COX-2-dependent overproduction of PGD_2_ increases quickly and dramatically in response to NSAIDs in patients with AERD, which is associated with other markers of mast cell activation such as tryptase [15,34,51,53]. The rapid response suggests the presence of chronically elevated intracellular baseline levels of pre-formed PGD_2_ resulting from the persistent hyperactivation of the COX pathway.

Although increased eosinophilia is a predominant histologic feature of nasal polyps and lower airways in subjects with AERD [6,14], recent studies using induced sputum revealed that neutrophilic, mixed eosinophilic/neutrophilic, and paucigranulocitic cell phenotypes are also frequently found in the same patients [57,58]. Using inflammatory patterns and the ratio between cysLTE4 and PGE2, three AERD subphenotypes were distinguished, associated with different levels of clinical asthma severity [57]. Moreover, non-eosinophilic phenotypes could also be classified into four subtypes using two demographic/clinical variables (age of the patients and asthma severity) and one metabolic variable (PGD2 level in induced sputum) [58]. The mechanisms involved in the pathogenesis of AERD with different inflammatory phenotypes and their potential used to predict response to classical and new therapies remain to be elucidated.

Human eosinophils can produce significant amounts of PGD_2_ catalysed by eosinophil-expressed H-PGDS [59]. H-PGDS levels have been shown to be elevated in nasal polyps and peripheral blood eosinophils of patients with AERD, which may contribute to the increased production of PGD_2_ at the baseline and after aspirin exposure [59].

In contrast to the numerous studies reporting increased PGD_2_ production by mast cells and eosinophils, very little is known about the production of PGD_2_ by structural cells such as airway epithelial cells and fibroblasts. To the best of our knowledge, only one study reported reduced PGD_2_ production by stimulated bronchial fibroblasts from patients with AERD compared to individuals with non-AERD and healthy controls [20]. This observation suggests that, as expected, the disturbed COX regulation in AERD nasal polyp fibroblasts results in a similarly deficient production of all prostanoids tested so far (PGE_2_ and PGD_2_), supporting the hypothesis that the downregulation of prostanoid metabolism reported in AERD is primarily localised in the airway epithelium, while inflammatory cells (mast cells and eosinophils) escape this anomaly.

### 3.5. Epithelial Cells, Alarmins, T2 Inflammation, and PGE_2_ in N-ERD

Recent research has described the complex composition of the epithelial barrier by the identification of different cell subsets: ciliated cells, goblet cells, submucosal glandular cells, secretory cells, basal cells (BCs), and some rare cells such as ionocytes and tuft cells [60]. The epithelial barrier, once thought to be just a physical shield, is now recognised as a key player in the regulation of immune responses in the airways of patients with asthma [61] and CRSwNP [62].

Environmental factors, such as pollutants, pathogens, and toxins, can activate the normal defensive response of the airway epithelium [61,62]. By mechanisms which remain to be fully elucidated, a disrupted epithelial structure can be the first step towards the development of asthma and CRSwNP resulting in an alarmin response (TSLP, IL-25, and IL-33) disproportionate to the inhaled triggers, which can further exacerbate the disruption of the airway epithelium [63]. The damaged barrier facilitates the penetration of pathogens and allergens, and, once recognised by pattern recognition receptors, it induces innate and adaptive immune responses [63]. Inhaled allergens are known to drive a T2 inflammatory response through the polarisation of T cells by dendritic cells mediated by TSLP [63]. Epithelial cells also recruit type 2 immune cells such as mast cells and eosinophils, which in turn release cytokines (IL-4, IL-5) and chemokines, contributing to activating group 2 innate lymphoid cells (ILC2s) and Th2 cells. These amplify and perpetuate the inflammatory loop underlying airway diseases such as asthma and CRSwNP [61,62,63].

Patients with asthma, especially those with severe asthma, have been found to exhibit increased levels of TSLP and T2 cytokines in the airways and ILC2s in their peripheral blood and BALF compared to healthy individuals [64,65,66]. Elevated ILC2 numbers have also been reported in nasal tissue from CRSwNP [67,68].

Recent studies have found new key regulatory functions of the airway epithelium, demonstrating that BCs have the capacity for inflammatory memory and self-renewal to maintain epithelial barrier integrity by the regeneration of other epithelial cells [69]. A greater number of BCs have been reported in the upper and lower airways in patients with CRSwNP and asthma. Interestingly, the BCs of patients with CRSwNP can acquire inflammatory memory with exposure to IL-4 and IL-13, which allows them to implement a fast and robust response against antigen re-stimulation [69,70,71].

Alarmins such as TSLP and IL-13 are elevated in the nasal polyps of patients with AERD [22,55]. In addition, in patients with AERD, the ILC2 numbers are significantly increased in nasal scrapings and decreased in the blood at the time of COX-1 inhibitor-induced airway reactions [68]. The number of ILC2s positively correlates with the levels of urinary LTE_4_ and PGD_2_ and symptom severity [68]. These findings suggest that the alarmin-mediating ILC2 expression axis may be involved in AERD pathogenesis.

Although the mechanism contributing to the inflammatory response in AERD appears to be complex, it is likely that epithelial cells, alarmins, mast cells, and ILC2s all participate in a feedback loop that further amplifies type 2 immunity [72].

Numerous observations support the notion that the PGE_2_-EP2/EP4 signalling axis exerts a key endogenous negative regulation of this feedback loop by a varied and complex complementary mechanism [73]. In vitro studies have found that exogenous PGE_2_ stabilises aspirin-induced inflammatory mediator release from eosinophils and mast cells [50,74]. Furthermore, recent studies support the involvement of PGE_2_ in the regulation of the IL-33/TSLP-ILC2 axis. PGE_2_ suppress TSLP expression and restricts ILC2 proliferation in response to IL-33 and TSLP, as well as their production of IL-5 and IL-13 [75]. Deficient EP2 expression in a genetically modified mouse enhanced lung ILC2 responses, while PGE_2_-EP2/EP4 axis signalling activation consistently reduced Th2 lung inflammation by the negative regulation of IL-2 responses [76]. IL-33 activates T2 innate inflammatory responses by signalling through the suppressor of tumorigenicity (ST2) cognate receptor, which in turn is regulated by the soluble ST2 receptor (sST2), a decoy receptor which neutralises excessive IL-33-induced T2 inflammation. A recent study reported the capacity of PGE_2_ to enhance the production of sST2 receptors by mast cells, thereby restricting IL-33 signalling through the ST2 receptor, limiting the severity of T2 adaptative immune responses [77].

**Figure 2:**
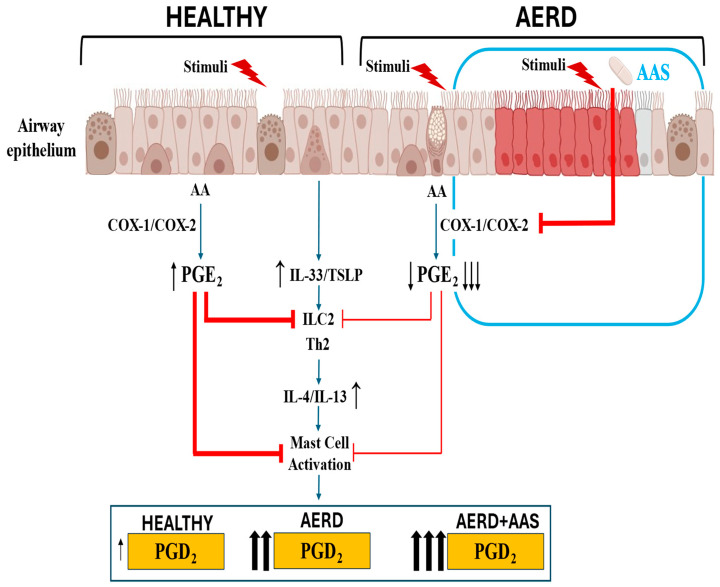
Response of airway epithelium to environmental triggers in subjects who are healthy or have AERD at the baseline and after aspirin exposure. In healthy airway epithelium, PGE2 modulates alarmin release, which is maintained within homeostatic limits. In AERD, decreased COX-2 expression and reduced PGE2 production by the disrupted epithelium facilitate excessive alarmin release, which in turn increases PGD2 production by mast cells. In patients with AERD, aspirin exposure further decreases PGE2 production, precipitating an acute increase in PGD2 by mast cells, contributing to the development of bronchoconstriction, nasal obstruction, and extra thoracic symptoms (urticaria, abdominal cramps).

All these findings on the crucial regulatory role played by the PGE-EP2 axis in airway inflammatory diseases, such as asthma and CRSwNP, support the notion that the low PGE_2_ production by the airway epithelium in AERD results in an ongoing activation of inflammatory cells, which in turn increases the release of pro-inflammatory cytokines and metabolites, such as PGD_2_ and Cys-LTs (Figure 2). Diminished PGE_2_ regulation in AERD leads to respiratory reactions upon COX-1 inhibition. Inhaled PGE_2_ prevents the bronchoconstriction associated with increased LT release in patients with AERD who have been challenged with aspirin [78]. These results strongly support the fact that PGE_2_ has a protective role in AERD pathogenesis.

## 4. Conclusions

In summary, research on AERD pathogenesis implies the following (Figure 2):

1. COX pathway dysregulation in the airway epithelium is an important factor contributing to the development of the disease;
2. Decreased PGE_2_ production by the respiratory epithelium leads to the development of an excessive innate immune response and the release of alarmins IL-33 and TSLP, which in turn activate the synthesis of pro-inflammatory metabolites, such as PGD_2_ by mast cells and eosinophils;
3. The link between these subsequent events can explain the disparate regulation at the baseline and after aspirin provocation of PGE_2_ and PGD_2_, prostanoids which are synthesised through the same COX pathway;
4. Regarding whether the airway epithelium holds the answer to the above paradox, the answer is affirmative, as the low production of PGE_2_ by airway cells can account for the increased production of PGD_2_ by mast cells and eosinophils;
5. A better understanding of the role of the airway epithelium would contribute to elucidating the mechanism underlying AERD.

## Abbreviations

AA	Arachidonic acid
AERD	Aspirin-exacerbated airway disease
BC	Basal cell
COX	Cyclooxygenase
CRS	Chronic rhinosinusitis
CRTH2	Chemoattractant receptor-like molecule expressed on Th2 cells
EP	PGE_2_ receptor
H-PGDS	Hematopoietic PGD2 synthase
mPGES-1	Microsomal PGE_2_ synthase-1
NP	Nasal polyp
NSAID	Nonsteroidal anti-inflammatory drug
PG	Prostaglandin
PLA2	Phospholipase A2
ST2	Suppressor of tumorigenicity 2
TSLP	Thymic stromal lymphopoietin
